# Early Cervical Lesions Affecting Ovarian Reserve and Reproductive Outcomes of Females in Assisted Reproductive Cycles

**DOI:** 10.3389/fonc.2022.761219

**Published:** 2022-03-21

**Authors:** Qiyu Yang, Juan Hu, Meng Wang, Zhou Li, Bo Huang, Lixia Zhu, Qingsong Xi, Lei Jin

**Affiliations:** ^1^ Reproductive Medicine Center, Tongji Hospital, Tongji Medical College, Huazhong University of Science and Technology, Wuhan, China; ^2^ Oncology Center, Tongji Hospital, Tongji Medical College, Huazhong University of Science and Technology, Wuhan, China

**Keywords:** cervical intraepithelial neoplasia, cervical cancer, ovarian reserve, assisted reproductive technology, cumulative live birth rate

## Abstract

To estimate the effects of early cervical lesions (ECL) on female reproductive function and IVF/ICSI cycle outcomes, a retrospective cohort study involving 111 infertile women from 2014 to 2019 was performed. Thirty-seven women with a history of ECL and seventy-four controls, undergoing IVF/ICSI cycles, were included in the ECL group and comparison group respectively. Demographic characteristics, ovarian reserve, and IVF/ICSI cycle outcomes of both groups were collected. Basal serum FSH level, AMH level, AFC, number of oocytes retrieved and matured, normal fertilization rate, embryo available rate, blastocyst formation rate, implantation rate, pregnancy rate, and cumulative live birth rate (CLBR) were assessed and compared. We observed that while both groups were similar concerning baseline features, significantly more women in the ECL group were diagnosed as poor ovarian response (POR), compared with those in the comparison group (27.0% *vs.* 10.8%, P=0.003). The pregnancy rate and LBR for a complete cycle were both significantly lower in the ECL group (38.5% *vs.* 58.8%, P=0.021; 28.9% *vs.* 48.2%, P=0.025, respectively). The conservative and optimal CLBRs for up to four complete cycles in the ECL group were also lower than those in the comparison group (40.5% *vs.* 55.4%, P=0.140; 45.9% *vs.* 67.6%, P=0.028). Longer time intervals (over one year) between ECL diagnosis/treatment and assisted reproductive technology (ART) cycle start negatively affected the pregnancy rate and LBR. In conclusion, female patients with ECL history seemingly have a lower ovarian reserve, reduced pregnancy rate, and decreased live birth rate (LBR), compared with age-matched women undergoing IVF/ICSI.

## Introduction

Cervical cancer remains the fourth most common cancer in women (1). With the development and popularization of screening methods, patients with cervical lesions can be early diagnosed and receive fertility-sparing treatment (2, 3). Cervical intraepithelial neoplasia (CIN), or squamous intraepithelial lesion (SIL), known as the pathological diagnosis of cervical precancerous lesion, was classified into low-grade lesion (CIN1) and high-grade lesions (CIN2 and CIN3), based on the different morphological characteristics and clinicopathological processes. CIN1 is attributed to active human papillomavirus (HPV) infection and usually regresses without treatment, while the high-grade SIL and early invasive cervical cancer, may need surgical treatments, such as conization, loop electrosurgical excision procedure (LEEP), and trachelectomy (4, 5).

The fecundability and obstetric outcomes of the patients with a history of early cervical lesions (ECL), including CIN and early invasive cancer, were analyzed previously. A case-control study showed a 2-fold increase in risk of infertility for women after CIN treatments compared with untreated women (6), while other studies involving larger samples demonstrated no evident adverse effects of CIN history and treatments on fertility (7, 8). As for early invasive cervical cancer, previous studies mainly focused on obstetric outcomes following different-type surgeries, such as abdominal, vaginal, or robot-assisted radical trachelectomy, aiming to prove the feasibility and safety of surgeries (9–12). Generally, most studies indicated that no significant negative effect of ECL and related surgical treatment on female fertility was observed.

But like the non-oncology population, even if the treatment of the cervix does not increase the prevalence of infertility, some patients are unable to conceive spontaneously for various reasons and require assisted reproductive technology (ART) to obtain offspring (7, 13). Whether the infertile patients with an ECL history would experience worse ART outcomes remains to be explored. In the current study, by retrospective analyses of the patients with ECL history undergoing ART in our center, the ovarian reserve, ovarian response, embryo development, and obstetrical outcomes were compared with those of the comparison group, to estimate the effect of ECL on female reproductive function and *in vitro* fertilization (IVF)/intracytoplasmic sperm injection (ICSI) outcomes.

## Materials and Methods

### Study Population

All women undergoing IVF/ICSI cycles from January 2014 to December 2019 at the Reproductive Medicine Center of Tongji Hospital in Wuhan, China were reviewed. Thirty-seven women who were noted to have a history of pathologically confirmed ECL were included in the ECL group. For each patient, two age-matched controls with similar body mass index (BMI) and infertility type during the same period were included in the comparison group. Women with other benign or malignant tumors, polycystic ovarian syndrome (PCOS), endometriosis, congenital abnormality, previous ovarian surgery, oocyte donors, and preimplantation genetic diagnosis cycles were excluded from the analyses in both groups.

The original study was approved by the Ethical Committee of Tongji Hospital, Tongji Medicine College, Huazhong University of Science and Technology on 24 April 2019 (#[2019]S964). Each of the patients had given written informed consent at the time of treatment for the future use of their clinical data.

### Ovarian Stimulation Protocol, Oocyte Retrieval, and Embryo Transfer

Ovarian stimulation protocols have been processed as previously described (14, 15). Briefly, pituitary suppression was achieved by injection of GnRH agonist starting in the mid-luteal phase of the previous cycle or GnRH antagonist starting with the existence of follicles measuring 13-14 mm in diameter. The dosage and duration of recombinant follicle-stimulating hormone (FSH) were adjusted based on individual ovarian responses. When two to three leading follicles reached a mean diameter of 18 mm, intramuscular injection of recombinant human chorionic gonadotropin (hCG) was performed, and oocytes were retrieved by guided transvaginal ultrasound 36-38h after hCG administration. The IVF or ICSI were performed as appropriate, and embryo transfer was performed on day 3 after oocyte retrieval. The surplus available embryos were frozen on day 3 or further cultured to day 5 or 6 for cryopreservation. Transfer with cryopreserved embryos was performed after priming the uterus with estrogen.

### Data Collection

Demographic characteristics, including age at cycle start, BMI, infertility duration, infertility type, and causes of infertility, were collected. Basal serum FSH level, antimüllerian hormone (AMH) level, and antral follicle count (AFC) were extracted for assessment of ovarian reserve. IVF/ICSI cycle information extracted included the amount of gonadotropin used, total days of ovarian stimulation, estradiol (E2) level, number of large follicles on hCG trigger day, number of oocytes retrieved and matured, available embryos, blastocysts, embryos transferred, pregnancies, and live births. For the ECL group, detailed information about cervical lesion type, treatment, and time from diagnosis/treatment of cervical lesion to cycle start was recorded.

### Criteria of Assessment

Women with poor ovarian response (POR) were classified with at least two of the three following features: advanced maternal age (≥40 years) or any other risk factor for POR; a previous POR (≤3 oocytes with a conventional ovarian stimulation protocol); and an abnormal ovarian reserve test (i.e. AFC <5–7 or AMH <0.5–1.1 ng/ml), according to the Bologna criteria (16). The normal fertilization rate was defined as the number of 2PN zygotes divided by the number of matured oocytes; the cleavage rate was defined as the number of cleaved embryos divided by the number of fertilized oocytes; the available embryo rate was defined as the number of embryos available for transfer, freezing, and extended culture divided by the number of normally-fertilized and cleaved embryos (including the late-cleaved embryos); the blastocyst formation rate was the number of blastocysts divided by the number of day 3 embryos for extended culture; the good-quality blastocyst formation rate was the blastocysts available for cryopreservation divided by the number of day 3 embryos for extended culture. Implantation rate referred to the ratio of the number of gestational sacs to the number of embryos transferred. Clinical pregnancy was confirmed if an intrauterine fetal heartbeat could be observed by transvaginal ultrasound. The live birth was defined as the birth of at least one live child after 28 weeks of gestation. Deliveries of multiple pregnancies were counted as one live birth.

For first-cycle cumulative live birth rate (CLBR) assessment, live birth rates (LBR) were calculated following every embryo-transfer procedure during the first complete cycle. For multiple cycles, two kinds of CLBRs were calculated. The conservative CLBR was calculated based on the assumption that women who discontinued ART treatment would not have achieved live birth if they had continued, while the optimal CLBR assumed that women who discontinued treatment would have had the same chance of live birth with continued ART as those who did continue (17). Women were considered to have discontinued ART treatment if they failed to have a treatment-dependent live birth and did not return for further ART cycles until 31 December 2019.

### Statistical Analysis

Data were analyzed and presented using SPSS software (SPSS Inc, version 22.0) and Graphpad Prism (version 8.0). Continuous data were analyzed with Kruskal-Wallis nonparametric method and expressed as median (interquartile range [IQR]), or with a Student t-test if data were normally distributed. Categorical data were presented as the number of cases and frequency (percentage), with a Chi-Square test to assess between-group differences. Logistic regression was performed to explore the influencing factors of cycle outcomes. The conservative CLBR estimate was calculated as the number of live births up to and including a specific treatment cycle, divided by the number of women who started their first ART cycle during the study period. The optimal estimate of CLBR was calculated by the Kaplan-Meier method upon inclusion of all treatment cycles in the analysis. Log-rank test and Kaplan-Meier curves with live birth considered as an event were used to illustrate differences between groups (14). Wald P-values were two-sided; P<0.05 was considered to be significant.

## Results

### Baseline Characteristics

Thirty-seven women with a history of ECL, involving fifty-three IVF/ICSI cycles, were identified and included in the ECL group. Age-matched women were included in the comparison group at a ratio of 1:2, with eighty-six IVF/ICSI cycles. The median age of women commencing ART in both groups was 34 years. Other baseline features, including BMI, infertility type and duration, and infertility cause, were also similar between the two groups (**Table 1**).

**Table 1:** Demographic characteristics of the ECL and comparison groups.

Characteristics	ECL patients	Comparison	P-value
Number of patients, n	37	74	/
Number of ART cycles, n	53	86	/
Female age at cycle start (y)	34 (31–39)	34 (31-39)	0.712
BMI (kg/m^2^)	21.8 (19.8-23.4)	21.5 (19.8-23.3)	0.700
Infertility duration (y)	3 (1-5)	3 (2-4.3)	0.659
Infertility type, n (%)			0.841
Primary	16 (43.2%)	30 (40.5%)	
Secondary	21 (56.8%)	44 (59.5%)	
Infertility cause, n (%)			0.733
Female factors only	26 (70.3%)	46 (62.2%)	
Tubal	16 (43.2%)	33 (44.6%)	
DOR	5 (13.5%)	11 (14.9%)	
Tubal +DOR	5 (13.5%)	2 (2.7%)	
Male factors only	2 (5.4%)	7 (9.5%)	
Combined male and female factors	4 (10.8%)	12 (16.2%)	
Unexplained	5 (13.5%)	9 (12.2%)	

Values are median (IQR) for continuous variables and n (%) for categorical variables.

ECL, early cervical lesions; ART, assisted reproductive technology; BMI, body mass index; DOR, diminished ovarian reserve.

Among the 37 patients with ECL history, 31 (83.8%) have been diagnosed as CIN and others as cervical cancer, based on pathological evidence. Four CIN1 patients and two CIN2 patients have received no treatment but regular follow-up, and three patients with CIN3 or early-stage cancer have undergone trachelectomy, with different extents of resection. Most patients (75.7%) have been treated with conization or LEEP. All patients underwent the re-examination of HPV infection and Thinprep cytologic test (TCT) regularly after cervical surgeries and specially prior to ART treatment, to exclude recurrence. Nearly half of the patients (45.9%) started the ART cycles within one year after ECL diagnosis or treatment, while 29.7% did not choose ART until two years later (**Supplementary Table S1**).

### Ovarian Reserve and Response

According to our observation, the ECL group had higher basal FSH level (median: 8.9 *vs.* 7.3 mIU/mL, P<0.001), lower AMH level (2.0 *vs.* 3.2 ng/mL, P=0.009), and fewer AFC (8.0 *vs.* 11.0, P<0.001) than the comparison group (**Table 2**). After stimulation, the ECL group reached significantly lower E2 levels and fewer large follicles on hCG trigger days, consequently fewer oocytes were obtained (6.0 *vs.* 9.0, P=0.015), although the oocyte maturation rates were similar between the two groups. Based on the Bologna criteria, more women with a history of ECL were diagnosed as POR compared with those without cervical lesion history (27.0% *vs.* 10.8%, P=0.003).

**Table 2:** Ovarian reserve and response to stimulation in the ECL and comparison groups.

Reproductive results	ECL patients	Comparison	P-value
Ovarian reserve			
Day3 FSH (mIU/mL)	8.9 (7.0-13.2)	7.3 (6.1-8.4)	**<0.001**
Day3 AFC	8.0 (4.0-10.0)	11.0 (7.0-16.0)	**<0.001**
AMH (ng/mL)	2.0 (1.1-4.0)	3.2 (1.8-5.8)	**0.009**
Ovarian response			
Total dose of gonadotropins (IU)	2400.0 (1875.5-3075.0)	2437.5 (1887.5-3018.8)	0.919
Days of gonadotropins use (d)	9.0 (8.0-10.5)	10.0 (8.8-11.0)	0.150
E2 on hCG trigger day (pg/mL)	1454.0 (694.4- 2329.5)	2084.0 (1356.5-3518.5)	**0.001**
No. of large follicles on hCG day	6 (4-10)	8 (6-13)	**0.008**
No. of oocytes retrieved	6 (3-11)	9 (5-15)	**0.015**
No. of MII oocytes	5 (3-8)	8 (4-13)	**0.021**
Maturation rate	89.1%	85.7%	0.213
Incidence of POR	27.0%	10.8%	**0.003**

ECL, early cervical lesions; FSH, follicle-stimulating hormone; AFC, antral follicle count; AMH, antimüllerian hormone; E2, estradiol; hCG, human chorionic gonadotropin; POR, poor ovarian response.

Bold fonts were statistically significant.

### IVF/ICSI Results, Obstetric and Neonatal Outcomes

As for the IVF/ICSI results, no significant differences were observed in normal fertilization rate, cleavage rate, available embryo rate, blastocyst formation rate, and good-quality blastocyst formation rate between the two groups. For fresh-embryo transfer cycles, the average numbers of transferred embryos were 1.26 and 1.41 in the ECL and comparison group, respectively (P>0.05). However, we found that the implantation rate, pregnancy rate, and LBR following fresh embryo transfer in the ECL group were all significantly lower than those in the comparison group (20.7% *vs.* 53.9%, P=0.002; 26.1% *vs.* 61.1%, P=0.005; 21.7% *vs.* 50.0%, P=0.021, respectively).

All pregnant women were followed up until a live birth was achieved or abortion occurred after their last IVF/ICSI cycles. For one complete cycle, which encompasses the outcomes from fresh and all frozen/thawed embryo transfers following one ovarian stimulation, the pregnancy rate and LBR of the ECL group were also significantly lower than those of the comparison group (38.5% *vs.* 58.8%, P=0.021; 28.9% *vs.* 48.2%, P=0.025, respectively) (**Table 3**).

**Table 3:** IVF/ICSI results and obstetric outcomes of ECL and comparison groups.

IVF/ICSI outcomes		ECL patients	Comparison	P-value
Normal fertilization rate		74.0% (267/361)	73.1% (542/741)	0.302
Cleavage rate		97.3% (323/332)	97.2% (649/668)	0.904
Available embryo rate		85.0% (243/286)	87.4% (514/588)	0.318
Blastocyst formation rate		73.6% (131/178)	69.5% (285/410)	0.560
Good-quality blastocyst formation rate		43.3% (77/178)	44.6% (183/410)	0.797
Fresh embryo transfer (ET)				
No. of fresh ET cycles		23	54	
Average no. of fresh embryos transferred		1.26	1.41	0.211
Implantation rate in fresh ET cycles		20.7% (6/29)	53.9% (41/76)	**0.002**
Pregnancy rate in fresh ET cycles		26.1% (6/23)	61.1% (33/54)	**0.005**
Single ET cycles		23.5% (4/17)	56.3% (18/32)	**0.028**
Double ET cycles		33.3% (2/6)	68.2% (15/22)	0.128
Live birth rate in fresh ET cycles		21.7% (5/23)	50.0% (27/54)	**0.021**
Pregnancy rate per complete cycle		38.5% (20/52)	58.8% (50/85)	**0.021**
LBR per complete cycle		28.9% (15/52)	48.2% (41/85)	**0.025**
Obstetric outcomes				
Preterm birth rate	Single ET cycle	0% (0/12)	0% (0/22)	1.000
	Double ET cycle	33.3% (1/3)	42.1% (8/19)	0.779
Newborns weight (g)		3347.5± 511.3	2998.1± 668.9	0.060
Maternal complications, n				/
Gestational diabetes		1	2	
Placental abnormalities		1	2	
Hypertensive disorder		0	1	
Neonatal defects		0	0	

ECL, early cervical lesions; LBR, live birth rate. Bold fonts were statistically significant.

**Figure 1:**
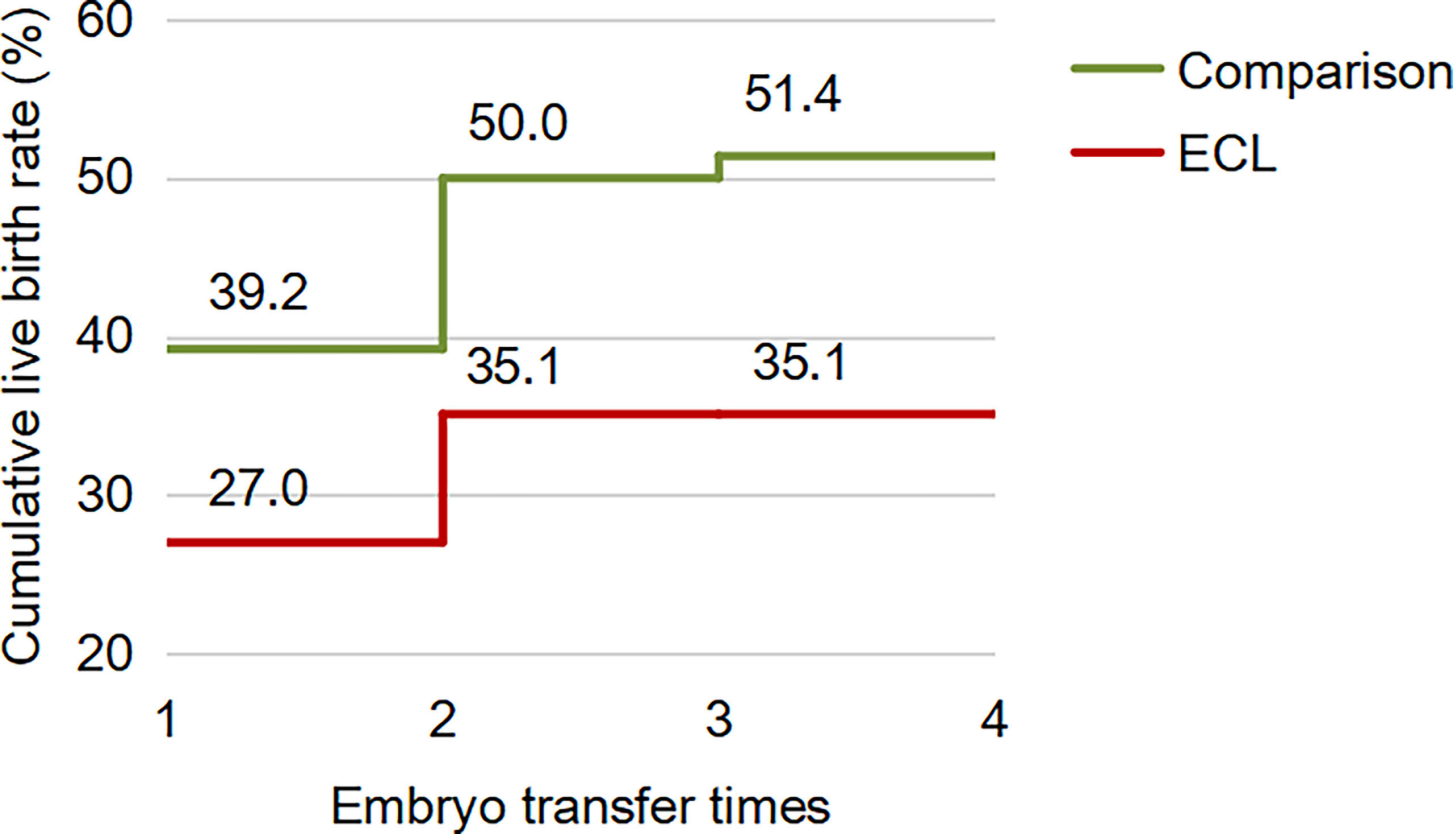
The CLBR following every embryo-transfer procedure for the first complete cycle. For each complete cycle, the LBR following every embryo-transfer procedure rose from 27.0% to 35.1% in the ECL group (red line), and from 39.2% to 51.4% in the comparison group (green line). The difference between the two groups was not significant (P=0.113).

**Figure 2:**
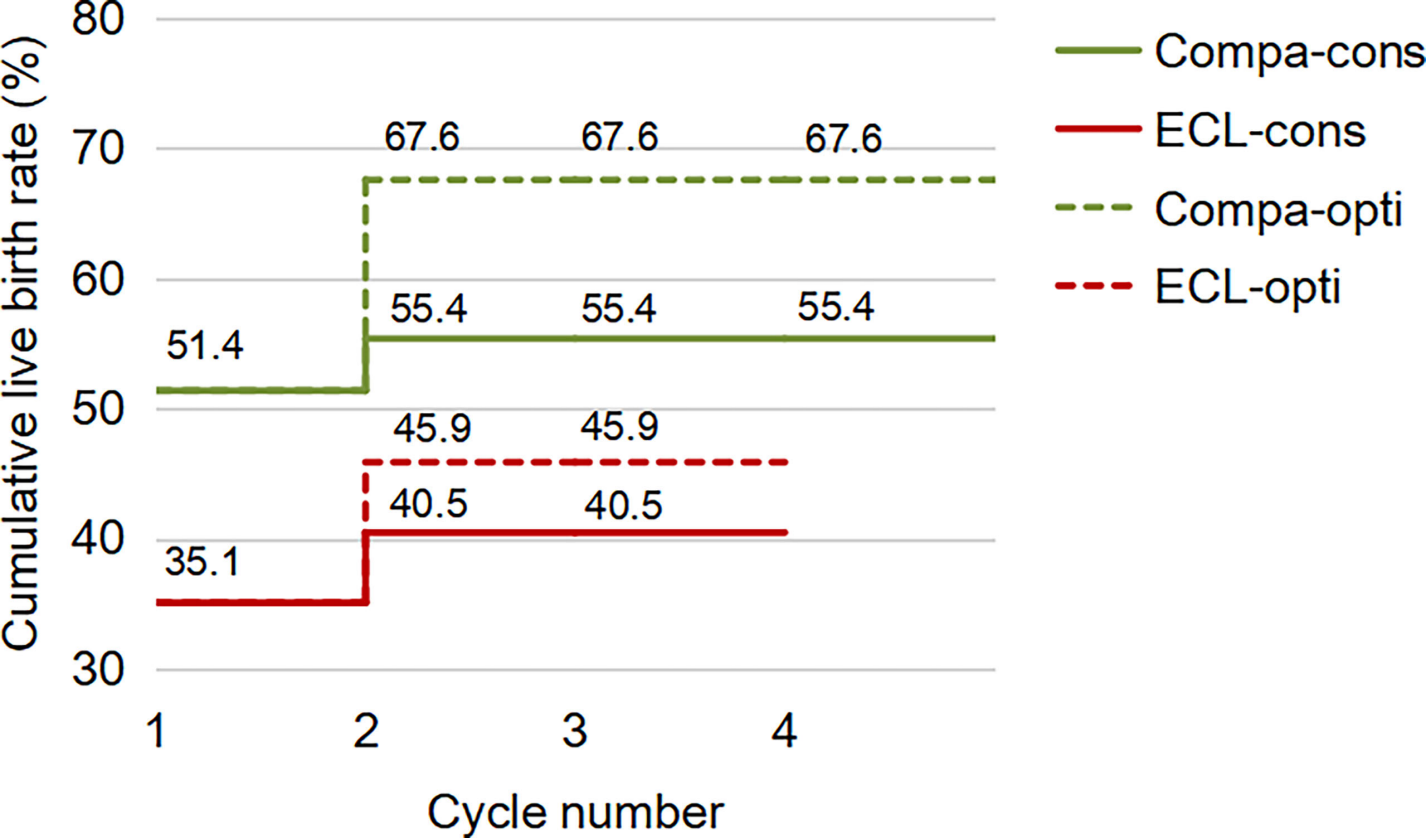
The conservative and optimal CLBRs for up to four complete cycles in both groups. The CLBR was 35.1% for the first complete cycle of the ECL group, rising to 40.5% (conservative) and 45.9% (optimal) for the second cycle. In the comparison group, the CLBR rose from 51.4% for the first cycle to 55.4% (conservative) and 67.6% (optimal) for the second cycle. The difference of optimal CLBRs between the two groups was significant (P=0.033), although not significant for conservative CLBRs (P=0.139). CLBRs did not increase from the third cycle in either group.

For the first complete cycle, the CLBR following every embryo-transfer procedure rose from 27.0% to 35.1% in the ECL group, and from 39.2% to 51.4% in the comparison group (P=0.113), shown in **Figure 1**. The conservative and optimal CLBRs for up to four complete cycles were presented in **Figure 2**. Overall, the CLBR was 35.1% for the first complete cycle of the ECL group, rising to 40.5% (conservative) and 45.9% (optimal) for the second cycle, while in the comparison group, the CLBR rose from 51.4% for the first cycle to 55.4% (conservative) and 67.6% (optimal) for the second cycle. The difference of optimal CLBRs between the two groups was significant (P=0.033). CLBRs did not increase from the third cycle in either group.

Considering the significantly lower pregnancy rate and LBR in the ECL group, factors such as age, BMI, lesion type, treatment, and the time interval between ECL diagnosis/treatment and IVF/ICSI cycle, were included in multivariate logistic regression for analysis (**Supplementary Table S2**). The results showed that higher BMI negatively affected the pregnancy rate but not the LBR. The pregnancy rate and LBR were both significantly reduced in patients with a time interval of more than one year between ECL diagnosis/treatment and IVF/ICSI, compared with the ones who started cycles within one year (pregnancy rate: 45.0% *vs.* 65.7%, OR: 0.087 [0.008-0.963], P=0.047; LBR: 30.0% *vs.* 52.9%, OR: 0.022 [0.001-0.623], P=0.027), although the median ages at cycle start were similar (33.5 *vs.* 34.0 years).

Of all the live births obtained by single embryo transfer in both groups, none were preterm delivery. All preterm births occurred during the course of twin pregnancies, and the incidence of preterm births and neonatal weight did not differ significantly between the two groups. Very few women in either group have maternal complications, including gestational diabetes, hypertensive disorder, and placental abnormalities. There were no cases of neonatal defects in both groups (**Table 3**).

## Discussion

As mentioned before, nowadays cervical lesions could be detected in early stage, and the risk of pre-invasive to invasive lesions shift might be predicted, based on the evaluation of some biomarkers, such as p16^ink^4a, p16, E-cadherin, Ki67, pRb, and p53 (18). Moreover, through the application of sentinel lymph node (SLN) mapping, the surgical treatment of cervical lesions also became more accurate (19). All these advances in technologies and concepts dramatically increased the fertility sparing opportunity for patients with cervical lesions, and also highlighted the necessity of reproductive evaluation and fertility guidance for these patients.

The results of this study suggest that female patients with CIN or early cervical cancer history have lower ovarian reserve, reduced pregnancy rate, and decreased CLBR, compared with age-matched women undergoing IVF/ICSI. Although the infertility rate and IVF delivery proportion of patients with ECL history were not increased according to previous studies (7, 8), it appears that those infertile patients do not have an optimal outcome in seeking ART treatment. Moreover, the time interval between ECL diagnosis/treatment and ART cycle start is seemingly associated with the pregnancy rate and LBR.

The deleterious impact of ECL history on the ovarian reserve is unexpected because lesions were localized and all treatments did not seem to directly involve the ovaries. The underlying mechanisms probably involve cervical treatments, HPV infection, and the potential reproductive damaging effects of the tumor itself:

The various cervical treatments reportedly caused different pregnancy results (20), while very little literature investigated their effects on ovarian reserve. Spracklen et al. reported that women with a history of LEEP required more time to conceive a pregnancy resulting in live birth when compared to similar women with no history of cervical surgery (6), but Sklavos et al., attempting to clarify the mechanism, found that the delayed time to pregnancy is likely not due to a LEEP-associated decrease in ovarian reserve as measured by AMH (21). Another study involving eighteen patients undergoing abdominal radical trachelectomy found no significant differences in AMH levels between surgery group and control group (22). However, the conclusions require further verification due to the small sample size. In the current study, the effect of cervical treatments was not evaluated specifically considering the limited number of untreated patients and possible inconsistency in surgical operations.

As for HPV infection, the majority in the ECL group (67.6%) were with clear evidence of HPV positive when they were diagnosed as ECL, but more information regarding HPV types was regrettably missing. A most recent study investigated the long-term impact of being diagnosed with high risk (HR)-HPV-positive and HR-HPV-negative lesions in a large group of women treated with conization for high-grade cervical dysplasia, and found that HR-HPV-positive patients experienced a 8-fold increase risk of recurrence than HR-HPV-negative counterpart (23). We speculated the HR-HPV infection might have a more sustained impact on patients. However, there is a lack of studies regarding HPV effects on female hormones or oocyte production, while more attention has been focused on the impact of HPV vaccines on ovarian reserve (24). Although a causal conclusion cannot be confirmed by now, six case reports indicated a possible association between the 4-valent HPV vaccine and primary ovarian insufficiency (POI) (25, 26). We hypothesized that HPV, which contains more active biological substances than its vaccine, may be more likely to be detrimental to ovarian function. More research regarding the long-term effect of HPV infection or 2-, 4-, 9-valent vaccines on ovarian reserve is required.

Additionally, the impact of the tumor itself on reproductive function should not be ignored. A meta-analysis reviewed seven studies that evaluated the ovarian performance of patients with cancers, including breast cancer, lymphoma or leukemia, gynecologic cancer, or other malignancies. The result showed that, even before radio/chemotherapy, the number of retrieved oocytes among patients with cancer was significantly lower compared with age-matched healthy IVF patients (27). In a recent study exploring the impact of cancer type on ovarian response to stimulation for fertility preservation, a lower ovarian response in patients with gynecologic cancers was identified (28). However, the conclusion remains controversial, based on the conflicting results of other studies (29, 30), and more studies are also needed to reveal the mechanisms.

Simultaneously, the ART outcomes of ECL patients in our study were also unsatisfactory. Two kinds of CLBRs in the ECL group were lower than those in the comparison group, with a statistical significance in optimal CLBRs difference. The conservative CLBR is pessimistic, while the optimal CLBR is probably overly optimistic, based on their computational principles. As the prognosis-adjusted CLBRs were closer to the optimal than the conservative estimate (17), we have more reason to believe that the ECL group did suffer from a lower CLBR.

HPV infection may be involved. Spandorfer et al. have reported significantly decreased pregnancy rates in IVF cycles in women with cervical HPV infection (without cytological abnormalities) who were undergoing IVF compared with those who were HPV negative (23.0% *vs.* 57.0%, respectively), and no significant difference in the miscarriage rates was found (31). Another study of 199 infertile couples, however, showed a marked increase in the risk of pregnancy loss when HPV infection was diagnosed (32). Importantly, a cervical lesion requiring treatment is more complex than a simple HPV infection, and the different treatments could affect ART outcomes, as shortened cervical length and abnormal cervical function are likely to increase the risk of miscarriage or preterm delivery (7, 33). The current study firstly presented a reduced pregnancy rate and CLBR in patients with cervical lesions undergoing ART treatment, while the impact on preterm delivery rate was not significant.

Our study took the ovarian reserve of ECL patients into consideration, which has usually been ignored by other researchers assessing the effect of HPV or cervical treatment on ART outcomes. In addition, the CLBRs were calculated both conservatively and optimistically to present the ART outcomes that patients are most concerned about. Limitations of this study include its retrospective and single-center nature, with a rather small sample size. The influence of treatment methods was not assessed specifically as the patients received surgeries in different centers.

In conclusion, we have shown that a history of early cervical lesion among infertile women may have a significantly negative impact on the ovarian reserve and IVF/ICSI outcomes, with a higher incidence of POR and lower live birth rate. This study provides data support for further exploration of the effects of cervical cancer and other tumors on fertility and also advocates that oncologists and reproductive physicians pay more attention to these patients. Comprehensive ovarian function assessment, individualized fertility guidance, and planned follow-up by professionals are recommended for patients with early cervical lesions history.

## Data Availability Statement

The original contributions presented in the study are included in the article/**Supplementary Material**. Further inquiries can be directed to the corresponding authors.

## Author Contributions

QY, JH, MW, QX, and LZ collected the clinical data. QY, LZ, ZL, and BH analyzed the data. QY, LZ, and MW composed the manuscript. QX and LJ were responsible for the concept and study design. All authors contributed to the interpretation, discussion and editing of the manuscript. All authors contributed to the article and approved the submitted version.

## Funding

This work was supported by the research grants from National Key Research and Development Project (2018YFC1002103) and the Health Commission of Hubei Province Scientific Research Project (WJ2021M110).

## Conflict of Interest

The authors declare that the research was conducted in the absence of any commercial or financial relationships that could be construed as a potential conflict of interest.

## Publisher’s Note

All claims expressed in this article are solely those of the authors and do not necessarily represent those of their affiliated organizations, or those of the publisher, the editors and the reviewers. Any product that may be evaluated in this article, or claim that may be made by its manufacturer, is not guaranteed or endorsed by the publisher.

## References

[1] Arbyn M Weiderpass E Bruni L de Sanjosé S Saraiya M Ferlay J. Estimates of Incidence and Mortality of Cervical Cancer in 2018: A Worldwide Analysis. Lancet Glob Health (2020) 8:e191–203. doi: 10.1016/S2214-109X(19)30482-6 PMC7025157 31812369

[2] Fontham ETH Wolf AMD Church TR Etzioni R Flowers CR Herzig A. Cervical Cancer Screening for Individuals at Average Risk: 2020 Guideline Update From the American Cancer Society. CA Cancer J Clin (2020) 70:321–46. doi: 10.3322/caac.21628 32729638

[3] Curry SJ Krist AH Owens DK Barry MJ Caughey AB Davidson KW. Screening for Cervical Cancer: US Preventive Services Task Force Recommendation Statement. JAMA (2018) 320:674–86. doi: 10.1001/jama.2018.10897 30140884

[4] Schiffman M Doorbar J Wentzensen N de Sanjosé S Fakhry C Monk BJ. Carcinogenic Human Papillomavirus Infection. Nat Rev Dis Primers (2016) 2:16086. doi: 10.1038/nrdp.2016.86 27905473

[5] Souho T Benlemlih M Bennani B. Human Papillomavirus Infection and Fertility Alteration: A Systematic Review. PloS One (2015) 10:e0126936. doi: 10.1371/journal.pone.0126936 25992782 PMC4436317

[6] Spracklen CN Harland KK Stegmann BJ Saftlas AF. Cervical Surgery for Cervical Intraepithelial Neoplasia and Prolonged Time to Conception of a Live Birth: A Case-Control Study. BJOG (2013) 120:960–5. doi: 10.1111/1471-0528.12209 PMC3691952 23489374

[7] Jakobsson M Gissler M Tiitinen A Paavonen J Tapper AM. Treatment for Cervical Intraepithelial Neoplasia and Subsequent IVF Deliveries. Hum Reprod (2008) 23:2252–5. doi: 10.1093/humrep/den271 18635529

[8] Wise LA Willis SK Perkins RB Wesselink AK Klann A Crowe HM. A Prospective Study of Treatments for Cervical Intraepithelial Neoplasia and Fecundability. Am J Obstet Gynecol (2019) 223:96.e1–15. doi: 10.1016/j.ajog.2019.12.017 PMC8041134 31887271

[9] Nishio H Fujii T Sugiyama J Kuji N Tanaka M Hamatani T. Reproductive and Obstetric Outcomes After Radical Abdominal Trachelectomy for Early-Stage Cervical Cancer in a Series of 31 Pregnancies. Hum Reprod (2013) 28:1793–8. doi: 10.1093/humrep/det118 23624633

[10] Johansen G Lönnerfors C Falconer H Persson J. Reproductive and Oncologic Outcome Following Robot-Assisted Laparoscopic Radical Trachelectomy for Early Stage Cervical Cancer. Gynecol Oncol (2016) 141:160–5. doi: 10.1016/j.ygyno.2016.01.028 26845228

[11] Li X Xia L Li J Chen X Ju X Wu X. Reproductive and Obstetric Outcomes After Abdominal Radical Trachelectomy (ART) for Patients With Early-Stage Cervical Cancers in Fudan, China. Gynecol Oncol (2020) 157:418–22. doi: 10.1016/j.ygyno.2020.02.016 32122687

[12] Shinkai S Ishioka S Mariya T Fujibe Y Kim M Someya M. Pregnancies After Vaginal Radical Trachelectomy (RT) in Patients With Early Invasive Uterine Cervical Cancer: Results From a Single Institute. BMC Pregnancy Childbirth (2020) 20:248. doi: 10.1186/s12884-020-02949-1 32334568 PMC7183613

[13] Nezhat C Roman RA Rambhatla A Nezhat F. Reproductive and Oncologic Outcomes After Fertility-Sparing Surgery for Early Stage Cervical Cancer: A Systematic Review. Fertil Steril (2020) 113:685–703. doi: 10.1016/j.fertnstert.2020.02.003 32228873

[14] Xu B Chen Y Geerts D Yue J Li Z Zhu G. Cumulative Live Birth Rates in More Than 3,000 Patients With Poor Ovarian Response: A 15-Year Survey of Final *In Vitro* Fertilization Outcome. Fertil Steril (2018) 109:1051–9. doi: 10.1016/j.fertnstert.2018.02.001 29935642

[15] Geng Y Xun Y Hu S Lai Q Jin L. GnRH Antagonist Versus Follicular-Phase Single-Dose GnRH Agonist Protocol in Patients of Normal Ovarian Responses During Controlled Ovarian Stimulation. Gynecol Endocrinol (2019) 35:309–13. doi: 10.1080/09513590.2018.1528221 30430883

[16] Ferraretti AP La Marca A Fauser BC Tarlatzis B Nargund G Gianaroli L. ESHRE Working Group on Poor Ovarian Response Definition. ESHRE Consensus on the Definition of ‘Poor Response’ to Ovarian Stimulation for *In Vitro* Fertilization: The Bologna Criteria. Hum Reprod (2011) 26:1616–24. doi: 10.1093/humrep/der092 21505041

[17] Chambers GM Paul RC Harris K Fitzgerald O Boothroyd CV Rombauts L. Assisted Reproductive Technology in Australia and New Zealand: Cumulative Live Birth Rates as Measures of Success. Med J Aust (2017) 207:114–8. doi: 10.5694/mja16.01435 28764619

[18] Valenti G Vitale SG Tropea A Biondi A Laganà AS. Tumor Markers of Uterine Cervical Cancer: A New Scenario to Guide Surgical Practice? Updates Surg (2017) 69:441–9. doi: 10.1007/s13304-017-0491-3 28918603

[19] Rossetti D Vitale SG Tropea A Biondi A Laganà AS. New Procedures for the Identification of Sentinel Lymph Node: Shaping the Horizon of Future Management in Early Stage Uterine Cervical Cancer. Updates Surg (2017) 69:383–8. doi: 10.1007/s13304-017-0456-6 28466456

[20] Rob L Skapa P Robova H. Fertility-Sparing Surgery in Patients With Cervical Cancer. Lancet Oncol (2011) 12:192–200. doi: 10.1016/S1470-2045(10)70084-X 20619737

[21] Sklavos MM Spracklen CN Saftlas AF Pinto LA. Does Loop Electrosurgical Excision Procedure of the Uterine Cervix Affect Anti-Müllerian Hormone Levels? BioMed Res Int (2014) 2014:875438. doi: 10.1155/2014/875438 24707500 PMC3953513

[22] Muraji M Sudo T Iwasaki S Ueno S Wakahashi S Yamaguchi S. The Effect of Abdominal Radical Trachelectomy on Ovarian Reserve: Serial Changes in Serum Anti-Müllerian Hormone Levels. J Cancer (2012) 3:191–5. doi: 10.7150/jca.4316 PMC3354414 22606208

[23] Bogani G Sopracordevole F Di Donato V Ciavattini A Ghelardi A Lopez S. High-Risk HPV-Positive and -Negative High-Grade Cervical Dysplasia: Analysis of 5-Year Outcomes. Gynecol Oncol (2021) 161:173–8. doi: 10.1016/j.ygyno.2021.01.020 33514481

[24] Christianson MS Wodi P Talaat K Halsey N. Primary Ovarian Insufficiency and Human Papilloma Virus Vaccines: A Review of the Current Evidence. Am J Obstet Gynecol (2020) 222:239–44. doi: 10.1016/j.ajog.2019.08.045 31479634

[25] Little DT Ward HRG. Adolescent Premature Ovarian Insufficiency Following Human Papillomavirus Vaccination: A Case Series Seen in General Practice. J Investig Med High Impact Case Rep (2014) 2:2324709614556129. doi: 10.1016/j.ajog.2019.08.045 PMC4528880 26425627

[26] Colafrancesco S Perricone C Tomljenovic L Shoenfeld Y. Human Papilloma Virus Vaccine and Primary Ovarian Failure: Another Facet of the Autoimmune/Inflammatory Syndrome Induced by Adjuvants. Am J Reprod Immunol (2013) 70:309–16. doi: 10.1111/aji.12151 23902317

[27] Friedler S Koc O Gidoni Y Raziel A Ron-El R. Ovarian Response to Stimulation for Fertility Preservation in Women With Malignant Disease: A Systematic Review and Meta-Analysis. Fertil Steril (2012) 97:125–33. doi: 10.1016/j.fertnstert.2011.10.014 22078784

[28] Alvarez RM Ramanathan P. Fertility Preservation in Female Oncology Patients: The Influence of the Type of Cancer on Ovarian Stimulation Response. Hum Reprod (2018) 33:2051–9. doi: 10.1093/humrep/dew158 27370358

[29] Robertson AD Missmer SA Ginsburg ES. Embryo Yield After *In Vitro* Fertilization in Women Undergoing Embryo Banking for Fertility Preservation Before Chemotherapy. Fertil Steril (2011) 95:588–91. doi: 10.1016/j.fertnstert.2010.04.028 20542508

[30] Tulandi T Holzer H. Effects of Malignancies on the Gonadal Function. Fertil Steril (2012) 98:813–5. doi: 10.1016/j.fertnstert.2012.05.010 22633259

[31] Spandorfer SD Bongiovanni AM Fasioulotis S Rosenwaks Z Ledger WJ Witkin SS. Prevalence of Cervical Human Papillomavirus in Women Undergoing *In Vitro* Fertilization and Association With Outcome. Fertil Steril (2006) 86:765–7. doi: 10.1016/j.fertnstert.2006.01.051 16782096

[32] Perino A Giovannelli L Schillaci R Ruvolo G Fiorentino FP Alimondi P. Human Papillomavirus Infection in Couples Undergoing *In Vitro* Fertilization Procedures: Impact on Reproductive Outcomes. Fertil Steril (2011) 95:1845–8. doi: 10.1016/j.fertnstert.2010.11.047 21167483

[33] Pinborg A Ortoft G Loft A Rasmussen SC Ingerslev HJ. Cervical Conization Doubles the Risk of Preterm and Very Preterm Birth in Assisted Reproductive Technology Twin Pregnancies. Hum Reprod (2015) 30:197–204. doi: 10.1093/humrep/deu260 25358346

