# Supplementary material for: Early Cervical Lesions Affecting Ovarian Reserve and Reproductive Outcomes of Females in Assisted Reproductive Cycles
Source: Front Oncol. 2022 Mar 21;12:761219. doi: 10.3389/fonc.2022.761219 (PMC8979291; doi:10.3389/fonc.2022.761219)
Supplement: Supplementary file 1 [file Table_1.docx]

**Supplementary Table S1 Cervical lesion type and treatment history of the ECL group prior to ART**

| Patients (n=37) | Number (percentage) |
| --- | --- |
| Cervical lesion type | |
| CIN1 | 6 (16.2%) |
| CIN2 | 11 (29.7%) |
| CIN3 | 14 (37.8%) |
| Cancer (IA1-IB1 stage) | 6 (16.2%) |
| Treatment type | |
| None | 6 (16.2%) |
| Conization | 21 (56.8%) |
| LEEP | 7 (18.9%) |
| (Partial) Trachelectomy | 3 (8.1%) |
| Time since treatment or diagnosis (m) | |
| ＜12 | 17 (45.9%) |
| 12-23 | 9 (24.3%) |
| ≥24 | 11 (29.7%) |

*CIN*, cervical intraepithelial neoplasia; *LEEP*, loop electrosurgical excision procedure.

**Supplementary Table S2 Multivariate logistic regression results of influencing factors for pregnancy rate and LBR of patients with ECL history**

| Factors | Pregnancy rate | | | | | Live birth rate | | | | |
| --- | --- | --- | --- | --- | --- | --- | --- | --- | --- | --- |
|  | Wald | OR | 95%CI | | P values | Wald | OR | 95%CI | | P values |
|  |  |  | Lower | Upper |  |  |  | Lower | Upper |  |
| Female age at cycle start | 0.019 | 1.014 | 0.833 | 1.233 | NS | 1.212 | 0.287 | 0.064 | 1.288 | NS |
| BMI | 4.538 | 0.665 | 0.457 | 0.968 | **0.033** | 2.040 | 0.346 | 0.06 | 1.989 | NS |
| Lesion type | 0.367 | 1.442 | 0.528 | 3.941 | NS | 0.905 | 1.490 | 0.488 | 4.547 | NS |
| Treatment type | 2.966 | 0.716 | 0.234 | 2.193 | NS | 4.553 | 0.742 | 0.221 | 2.494 | NS |
| Time interval | 3.961 | 0.087 | 0.008 | 0.963 | **0.047** | 4.921 | 0.022 | 0.001 | 0.623 | **0.027** |

*ECL*, early cervical lesions; *BMI*, body mass index; *NS*, not significant.

Note: Age and BMI were included as continuous variables; lesion type and treatment type were classified as in Supplementary Table S1; time interval was coded as “1” for “<12 m” and “2” for “≥12 m”, for logistic regression.
